# Serum Anti-Müllerian Hormone Levels and Risk of Premature Ovarian Insufficiency in Female Childhood Cancer Survivors: Systematic Review and Network Meta-Analysis

**DOI:** 10.3390/cancers13246331

**Published:** 2021-12-16

**Authors:** Marco Torella, Gaetano Riemma, Pasquale De Franciscis, Marco La Verde, Nicola Colacurci

**Affiliations:** Obstetrics and Gynecology Unit, Department of Woman, Child and General and Specialized Surgery, University of Campania “Luigi Vanvitelli”, 80128 Naples, Italy; marco.torella@unicampania.it (M.T.); pasquale.defranciscis@unicampania.it (P.D.F.); marco.laverde@unicampania.it (M.L.V.); nicola.colacurci@unicampania.it (N.C.)

**Keywords:** childhood cancer survivors, anti-Müllerian hormone, ovarian reserve, fertility, premature ovarian insufficiency, radiotherapy, alkylating agents

## Abstract

**Simple Summary:**

Over the last twenty years, innovations in the treatment of childhood cancer have increased survival rates. However, female childhood cancer survivors (CCS) are prone to late reproductive aftereffects, including premature ovarian insufficiency (POI). Nonetheless, patients might experience different side effects on fertility according to the type of diagnosed cancer and subsequent treatment. Anti-Müllerian hormone (AMH) is currently used in reproductive medicine to screen for impaired ovarian reserves. However, it does not represent the gold standard in oncofertility. In this systematic review and network meta-analysis of age-matched case–control studies, we evaluate the role of AMH for ovarian reserve screening according to the type of childhood cancer and determined which group of survivors are more prone to POI by means of direct and indirect comparisons among the CCS cohorts.

**Abstract:**

Background: Female childhood cancer survivors (CCS) might have impaired ovarian reserves, especially after alkylating agents or radiotherapy. The purpose of this systematic review and network meta-analysis is to evaluate the role of serum anti-Müllerian hormone (AMH) for ovarian reserve screening and the risk of premature ovarian insufficiency (POI) according to the subtype of childhood cancer. (2) Methods: PRISMA-NMA guidelines were followed. We carried out a network meta-analysis based on a random effects model for mixed multiple treatment comparisons to rank childhood cancers effects on fertility by surface under the cumulative ranking curve (SUCRA). Studies were selected only if they had an age-matched control group. Quality assessment was performed using Newcastle–Ottawa Scale. The co-primary outcomes were mean AMH levels and the incidence of POI. (3) Results: A total of 8 studies (1303 participants) were included. Women treated for a neuroblastoma during infancy were more likely to be ranked first for impaired AMH levels (SUCRA = 65.4%), followed by mixed CCS (SUCRA = 29.6%). The greatest rates of POI were found in neuroblastoma survivors (SUCRA = 42.5%), followed by acute lymphoid leukemia (SUCRA = 26.3%) or any other neoplasia (SUCR A = 20.5%). (4) Conclusions: AMH represents a trustworthy approach for ovarian reserve screening. Direct and indirect comparisons found no differences in mean AMH levels and POI risk between subtypes of CCS and healthy controls. SUCRA analysis showed that female neuroblastoma survivors were more at risk for reduced serum AMH levels and increased risk of POI.

## 1. Introduction

Therapies for cancer experienced during infancy or adolescence are known to harm fertility due to direct and indirect damage to the ovaries, with a significant loss of the ovarian reserves [1]. Evidence has proven that alkylating agents and body irradiation should be considered the most dangerous treatments for their toxic potential on the ovaries [2,3,4]. Nonetheless, it is still unknown which exact toxic doses, chemotherapeutic schemes, or radiotherapy protocols should be considered the most harmful [5,6]. A critical long-term side effect in women who survived cancer during youth or adolescence is the boost in follicle loss with impaired ovarian function, leading to absent puberty or premature menopause [7]. For this reason, the term premature ovarian insufficiency (POI) depicts the clinical diagnosis of the cease of ovarian function before 40 years of age [8]. In addition to the extremely poor reproductive outcomes of these patients, they are bothered by menstrual disturbances, including amenorrhea or oligomenorrhea, due to increased FSH and lowered estradiol levels. Hence, estrogen deprivation exposes these women to an increased risk for osteoporosis, cardiovascular diseases, and metabolic syndrome [9,10].

Therefore, it is crucial to identify women at risk of developing POI to manage further complications. 

Different methods are being used in clinical practice to assess ovarian reserves: antral follicle count (AFC), ovarian volume, and serum levels of follicle stimulating hormone (FSH), estradiol, and inhibin B. FSH, estradiol, and inhibin B are all part of the feedback system, which makes their serum levels dependent on each other. The use of oral contraceptives and menopausal hormone therapy (MHT) leads to several difficulties in the interpretation of FSH and female sex steroids levels for gonadal status. In addition, the ovarian function needs to be significantly decreased to have a critical raise of FSH levels, which cannot be used as a sentinel for anticipating premature menopause [11,12]. Because AFC (the cumulative amount of the growing follicles between 2 and 10 mm in diameters from both the ovaries) seems to correlate well with the primordial follicle pool, this method is currently the most often preferred way to predict ovarian reserves [13]. However, performing an ultrasonographic AFC requires a transvaginal approach, which cannot be performed in patients who never had a sexual intercourse, and it is often refused by young females for fear of pain and discomfort [12].

For this reason, there was a need for a less invasive ovarian reserve marker, especially in young women who suffered from a childhood cancer.

Anti-Müllerian hormone (AMH) is secreted by granulosa cells of growing pre- and early antral ovarian follicles. It has been remarked as an indirect measure of the “ovarian reserves”, the available set of primordial follicles retrievable in both ovaries at any time from birth to menopause [14]. The serum levels of AMH, low during childhood, increase until a plateau zone during the mid-twenties; subsequently, they decline until the menopause. AMH is representative of ovarian reserves in every age group and its dosage does not depend on the phases of the menstrual cycle.

Although AMH has been certified as a valid marker for ovarian reserves, it is not still used as gold standard in follow-up programs for female childhood cancer survivors (CCS) [15].

To date, AMH has been used especially in the work-up of women undergoing in vitro fertilization [16,17]. However, recent trials show uncertainty regarding the decline of serum AMH in CCS relative to healthy controls. Some studies showed a decrease in these levels in CCS women. This might indicate that AMH levels could serve as a serum marker to screen for impaired ovarian reserves in female CCS. It has been well established that AMH correlates with AFC in healthy women for ovarian reserve screening; however, such correlation has not been yet confirmed for ovarian reserve screening in female CCS [18]. 

The aim of this systematic review is to evaluate the differences between AMH levels and risk of POI in women who survived cancer during childhood or adolescence, compared to healthy controls, and to establish whether there are differences among the subtypes of malignancies by means of direct and indirect comparisons.

## 2. Materials and Methods

This network meta-analysis was conducted in accordance with the *Cochrane Handbook for Systematic Reviews of Interventions* [19] and the methods outlined in Mbuagbaw et al. [20]. It followed the preferred reporting items for systematic reviews and meta-analyses (PRISMA) extension statement for network meta-analyses (PRISMA-NMA) (see Supplement File S1). It was registered in the International Prospective Register of Systematic Reviews (PROSPERO) database (CRD42021288395).

### 2.1. Data Sources and Eligibility Criteria

Electronic databases (EMBASE, MEDLINE, Scopus, Scielo.br, LILACS, and the Cochrane Library at the CENTRAL Register of Controlled Trials) were searched from the inception of each database to June 2021. Search terms used were a combination of text words and medical subject headings (MeSH), including: “Anti-Mullerian Hormone” or “Antimullerian Hormone” or “AMH” and “female cancer survivors” (see Supplementary Materials Table S1). CINAHL, PsycINFO, and AMED were also screened to find other relevant studies and reduce publication bias. To include additional evidence, Clinicaltrials.gov and the WHO International Clinical Trials Registry Platform (ICTRP) were also screened. Moreover, to retrieve conference abstracts of international and national congresses, the grey literature (NTIS, PsycEXTRA) was examined.

There were no restrictions for the geographic location or language of the original papers. In addition, the reference lists of all eligible studies were screened to assess studies not captured by electronic searches. The electronic search and the potential eligibility of the investigated studies were checked in an independent manner by two researchers (M.T. and G.R.). Any potential disagreement was resolved by discussion with a third reviewer (N.C.).

Inclusion criteria were the following: prospective cross-sectional or retrospective case–control studies that included CCS women that were screened for AMH levels and/or POI and compared to age-matched healthy controls. Studies were excluded if they did not consider a control group or did not report at least one outcome of interest or lacked follow-up records with incomplete data.

The abstraction forms were carefully built for this network meta-analysis. The key characteristics recorded included: patient descriptors, study duration, setting, details of treatment, features of the treatment, outcomes evaluated, features of the control group, results, and quality elements. 

All the abstracts were reviewed and classified by two researchers (G.R. and M.L.V.) independently. The agreement for possible relevance was reached by consensus; the same two researchers carried out a full-text evaluation of the selected papers and independently extracted relevant data concerning the trial characteristics and the outcomes of interest. All the inconsistencies were examined by the reviewers and consensus was reached by asking a third author (P.D.F.). If necessary, unpublished data was obtained by directly contacting the authors of the original studies whenever the study methodology indicated that other outcome data were recorded. 

### 2.2. Main Outcomes Measures

The first co-primary outcome of this network meta-analysis was the mean AMH levels, defined as the serum blood levels of AMH retrieved from the blood samples of female CCS and healthy controls during the early follicular phase (day 2 to 5) of the menstrual cycle or randomly in the case of prolonged amenorrhea (no menses for at least 6 months). The second co-primary outcome was the rate of POI in CCS and controls, defined as absence of menarche or premature depletion of ovarian follicles before 40 years of age.

### 2.3. Risk of Bias

In cohort studies eligible for this meta-analysis, the risk of bias in each of the available papers was assessed using the Newcastle–Ottawa Scale (NOS) criteria [21]. According to these criteria, the overall bias of the study is based on three major elements: selection of study cohorts, comparability of these groups, and ascertainment of the interested outcomes. The evaluation for the selection of a study involves the following criteria: assessment of the representativeness of the exposed group, selection of the non-exposed group, ascertainment of the exposure, and demonstration that outcome of interest was not likely to occur spontaneously at the start of the study. The comparability assessment was performed examining the similarity of cohorts based on the design or analysis. Furthermore, the ascertainment of the exposure is evaluated upon the methodology to determine the outcome of interest, duration, and adequacy of the follow-up. Applying the NOS criteria, a study can be awarded a maximum of one star for each numbered item within the Selection and Outcome categories, meanwhile two stars can be assigned for comparability. The quality assessment using such criteria can reach a maximum overall score of 9. The NOS quality assessment was independently carried out by three authors (M.T., M.L.V., and N.C.) Any disagreement was resolved by involving an additional author (P.D.F.). 

### 2.4. Data Extraction and Statistical Analysis

To establish comprehensive pooled estimates of the effects on ovarian reserve and the subsequent POI risk of the different treatments for the subtypes of childhood cancers, we performed a quantitative meta-analysis regarding respective parameters based on the Bayesian theorem. This statistical method evaluates both direct and indirect data from trials using a common comparator to obtain estimates of the effects according to the kind of treatment based on multiple intervention comparisons [19,20].

All data analysis and graphical renderings were performed using STATA version 14.1 (StataCorp, College Station, TX, USA). For the evaluated outcomes, to statistically confirm the overall consistency assumption among the networks of the meta-analysis, the command <network meta inconsistency> was used. Subsequently, for the local test on loop inconsistency, the SIDE (separating indirect from direct evidence)-splitting method, by means of the command <network sidesplit all> was executed. When there was no inconsistency in both the global and the local tests, the consistency model was accepted. In this case, the consistency assumption suggested that the direct and indirect comparisons showed significant results, and the differences between the data analysis and results were likely related only to the effects of the intervention or random-based errors.

The summary measures were reported as odds ratio (OR) for categorical variables, mean difference (MD) for continuous variables using a 95% of confidence interval (CI), and adopting the random effects model of Der Simonian and Laird. Higgins I-squared (I^2^) index was used for assessing potential heterogeneity. I^2^ degrees of 0%, 25%, 50%, and 75% were considered thresholds for absent, low, intermediate, and high heterogeneity, respectively. In the cases of significant heterogeneity, sensitivity analyses were carried out to understand relevant sources of heterogeneity.

The potential publication bias was quantified by means of the Egger’s test. Subsequently, a forest plot and prediction interval plot were constructed to compare the impact of the different cancers on women’s fertility and to rank them to define the superiority in terms of reduced ovarian reserve using a ranking plot (Surface Under the Cumulative Ranking curve Area (SUCRA)). The SUCRA is a numeric representation of the overall ranking, and it sums up each treatment effect by a percentage, ranging from 0 to 100%. The higher the SUCRA value and the closer to 100%, the higher the likelihood that a therapy is at the top rank or one of the top ranks as the most influential treatment; the closer the SUCRA value is to 0, the more likely that a therapy is of being placed at the bottom rank, or one of the bottom ranks, describing it as a less influential treatment [20].

## 3. Results

### 3.1. Study Selection

**Figure 1:**
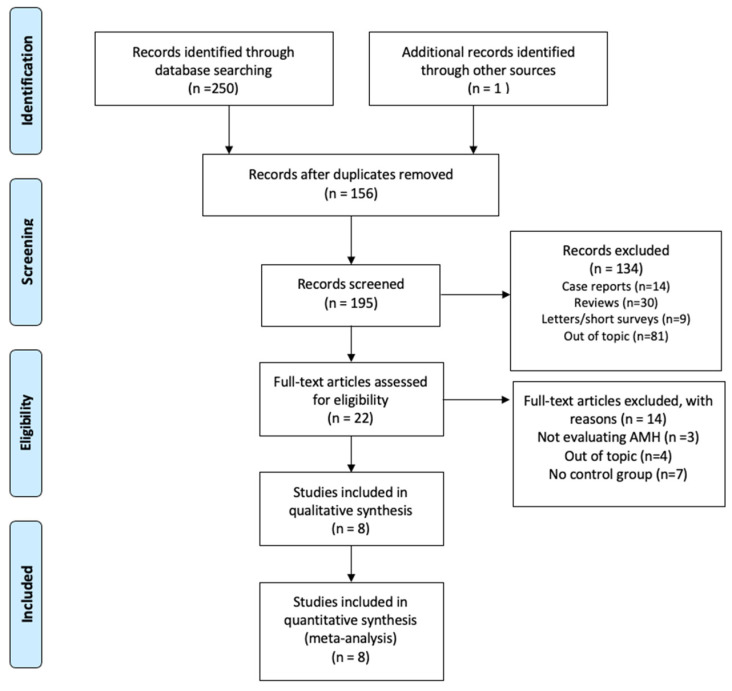
PRISMA flow diagram for the study selection.

A total of 251 studies were originally identified through the database searches. Of those, 95 were removed as duplicates. After title and abstract screening, 133 studies were subsequently removed (Figure 1). A total of 22 studies were assessed for full text, of which 3 were removed for not evaluating AMH levels, 4 were removed for being out of topic, and 7 trials were excluded because they did not include a control group. A total of 8 studies [8,22,23,24,25,26,27,28], including 1303 participants (663 female CCS and 640 age-matched healthy controls) were included in the quantitative synthesis and network meta-analysis (Figure 1). 

### 3.2. Study Characteristics

Women were followed in the included trials starting in 1964 and 2017. All studies included women who were diagnosed with solid or hematologic tumors during their childhood or adolescence. The studies were conducted in Europe and Asia. An age-matched control group was used for comparisons in every paper. All participants fully recovered from the pathology with no signs of relapse at the enrolment. Four studies compared an unselected cohort of female CCS to age-matched healthy controls [22,25,26,28]. Of those, Nystrom et al. [25] studied a mixed CCS population of women treated with different schemes of RT, without differences between groups, while Bath et al. [22], Van der Kooi et al. [28], and Harzif et al. [23] analyzed women subjected to CT or CT plus RT. One trial analyzed the effects on ovarian reserves of the treatment of a neuroblastoma [27], while Nies et al. [24] reported about survivors of differentiated thyroid cancer (Table 1).

Inclusion and exclusion criteria provided by cohort studies included in the network meta-analysis are reported in Table 2.

### 3.3. Quality Assessment 

Using the NOS criteria, all evaluated trials reported high scores, ranging from a minimum of 6 to a maximum of 9. The comparability of cohorts reached the maximum score based on controls for age as most important factor and based on BMI as the additional factor. The detailed point-by-point assessment is depicted in Supplementary Materials Table S2.

### 3.4. Synthesis of Results

#### 3.4.1. AMH Levels

Seven studies [8,22,23,24,25,27,28] compared the AMH levels in mixed (including hematologic, lymphoid, and solid tumors) CCS, neuroblastoma survivors, and controls to indirectly test the ovarian reserve.

**Figure 2:**
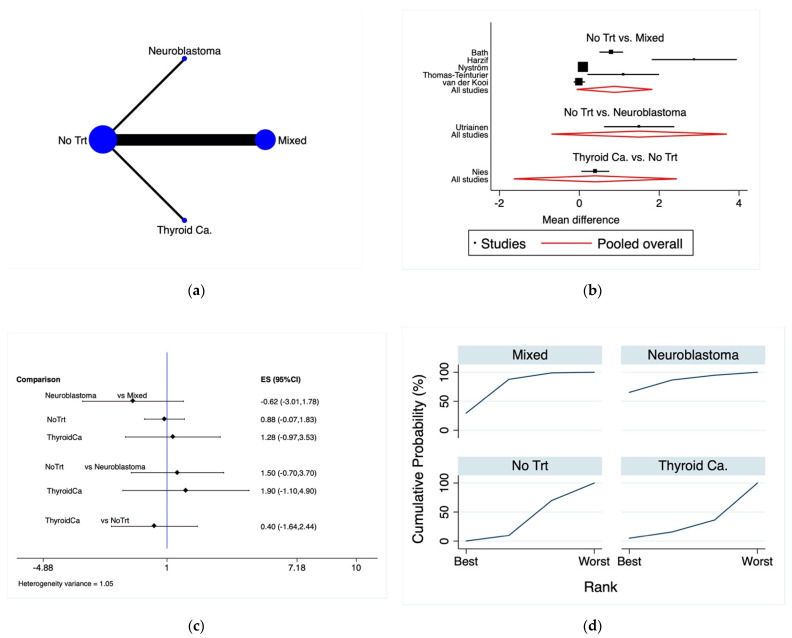
Mean anti-Mullerian hormone levels in female cancer survivors. (**a**) Network map of included studies: the lines indicate direct comparisons between groups, and the sizes of the areas of the circles indicate the respective sample sizes. (**b**) Forest plot; mean difference: a positive value favors the treatment on the right of the plot while a negative treatment favors the treatment on the left. Pooled overall: pooled results for each comparison. (**c**) Predictive interval plot. (**d**) SUCRA ranking plot. Abbreviations: ES: effect estimates; Mixed: unselected CCS population; NoTrt: no treatment; ThyroidCa: thyroid cancer.

Figure 2a shows the network of the included studies, with the frequency of studied population and the most accurate direct comparisons. The inconsistency analysis showed that global inconsistency was not retrievable (*p* = 0.416). 

The SIDE analysis showed that there were no substantial differences between direct and indirect comparisons in the closed loops considered for the network (local inconsistency) (see Supplementary Materials Table S3) and no significant differences between the consistency and inconsistency assumption models (*p* = 0.900). 

For this primary outcome, the Egger’s test (*p* = 0.211) indicated no significant publication bias in this network meta-analysis.

The forest plot (Figure 2b) and predictive interval plot (Figure 2c) revealed the influence of the CCS treatment on the reduction of the ovarian reserve measured by AMH levels among the networks. 

Concerning the mean differences between the AMH levels, no significant differences were found between survivors from an unselected population, neuroblastoma, and thyroid cancer relative to healthy controls (Table 3).

According to the SUCRA ranking plot (Figure 2d), women who were treated with a high dose radiotherapy for a neuroblastoma during infancy were more likely to have impaired AMH levels (SUCRA = 65.4%), followed by the mixed population (SUCRA = 29.6%), meanwhile women who survived a thyroid cancer and were treated with 131-I showed similar percentages to healthy controls (SUCRA = 4.9% and SUCRA = 0.1%, respectively).

#### 3.4.2. POI Incidence

**Figure 3:**
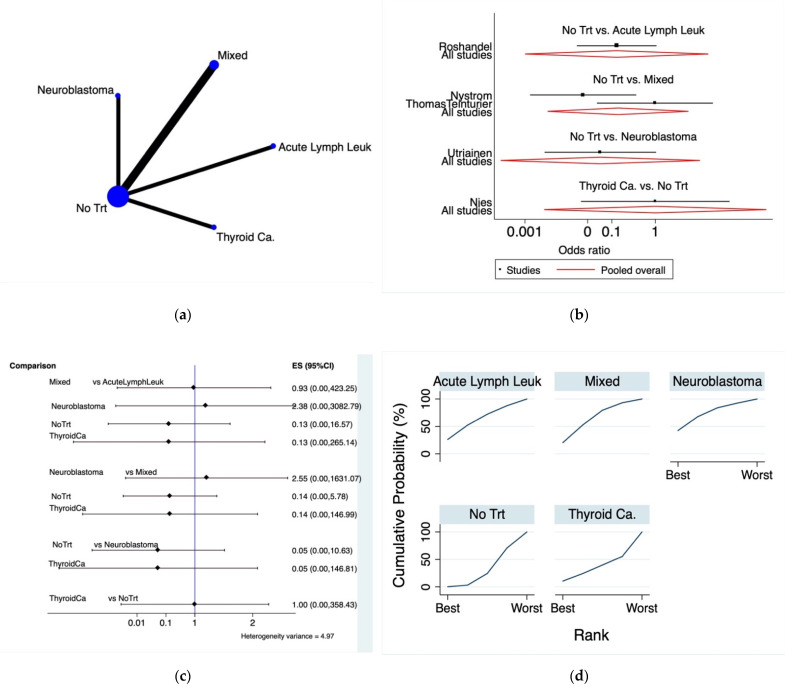
Premature ovarian insufficiency in female cancer survivors. (**a**) Network map of included studies: the lines indicate direct comparisons between groups, and the sizes of the areas of the circles indicate the respective sample sizes. (**b**) Forest plot: a positive value favors the treatment on the right of the plot while a negative treatment favors the treatment on the left. Pooled overall: pooled results for each comparison. (**c**) Predictive interval plot. (**d**) SUCRA ranking plot. Abbreviations: ES: effect estimates; AcuteLymphLeuk: acute lymphoid leukemia; Mixed: unselected CCS population; NoTrt: no treatment; ThyroidCa: thyroid cancer.

The incidence of POI among survivors and controls was analyzed in five cohort studies [8,24,25,26,27]. Neuroblastoma, acute lymphoid leukemia, thyroid cancer, or unselected female CCS were directly compared to healthy controls and indirectly compared between each other, as shown in the network map (Figure 3a). 

The SIDE analysis demonstrated no significant differences between direct and indirect comparisons in the closed loops analyzed in the network (local inconsistency) (see Supplementary Materials Table S4) with no significant changes between the consistency and inconsistency assumption models (*p* = 0.910). A publication bias judged by the Egger’s test (*p* = 0.476) was not apparent for this outcome.

The forest plot (Figure 3b) showed no statistical differences concerning the direct comparisons between the cancer survivors and age-matched controls for the mixed population, the neuroblastoma, thyroid cancer, and acute lymphoid leukemia women.

Analyzing the interval plot, there were no significant differences among the risk of POI between the different pathologies or healthy controls (Figure 3c).

Taking all together, according to the SUCRA ranking plot (Figure 3d), there were significant chances of being diagnosed with POI after treatment for neuroblastoma (SUCRA = 42.5%), followed by women treated for acute lymphoid leukemia (SUCRA = 26.3%) or for any other kind of neoplasia (SUCRA = 20.5%), while rare chances were retrievable for thyroid cancer (SUCRA = 1.9%) or no cancer (SUCRA = 1.6%).

## 4. Discussion

This systematic review and network meta-analysis of cohort studies showed that AMH is a reliable indirect way to evaluate the ovarian reserve in CCS. In women who survived a CCS or hematologic neoplasia, AMH levels do not show differences from the healthy population. However, healthy controls showed a reduced risk of POI relative to neuroblastoma survivors, and chances of being diagnosed with POI are higher in a mixed CCS population rather than in survivors of neuroblastoma, acute lymphoid leukemia, or thyroid cancer.

Gonadal function should be regularly tested during the long-term follow-up of female CCS [11,29]. However, it is difficult to evaluate ovarian function in childhood due to the nondiagnostic levels of serum gonadotropin and estradiol concentrations before the start of the puberty [30]. Therefore, the analysis of serum AMH levels is currently used to assess future and present fertility [31].

To date, AMH and AFC have shown the best trustworthiness as screening tests for ovarian reserves. However, a test with a 100% sensitivity or specificity for ovarian reserves does not exist [32,33]. In addition, for AFC, a transvaginal ultrasound probe is mandatory, giving additional discomfort. Moreover, younger women who did not have their first sexual intercourse cannot undergo the procedure [34]. Therefore, transvaginal sonography for AFC is still unavailable as a screening tool. Moreover, another advantage of AMH is that its levels do not change during the menstrual cycle, as opposed to inhibin B, whose values are the highest during the follicular phase. Compared to AMH, FSH raises as a late sign of diminished ovarian reserves, meanwhile estradiol levels are not considered trustworthy [7].

Previous studies have researched the fluctuations AMH levels before, during, and after treatment for cancer in young women [35]. In some girls, irrespective of age, there was a rapid decrease in AMH levels after the start of treatment. However, in those patients with a transient ovarian dysfunction, a rapid increase in AMH levels was highlighted up to two years after treatment. However, in some cases, the ovarian dysfunction could become unreversible, leading to POI [35]. For this reason, in the case of RT or alkylating agents, a strict follow-up and an oncofertility management (i.e., ovarian cryopreservation) is crucial to avoid future reproductive issues [36,37].

Concerning the effects of 131-I used for differentiated thyroid cancer, AMH levels in long-term follow-up women seem unaffected by the therapy [24]. Quantitatively, damages to the ovarian parenchyma caused by 131-I might be less hazardous in younger women since younger girls still have a satisfying number of primary oocytes/primordial follicles rather than adult women. As the number and quality of oocytes decrease with the increase in age, it has been seen that in women over 35 years of age who were treated with 131-I for DTC had a more significant impact on AMH levels and pregnancy rates, similarly to other CCS [38].

Although Nies et al. [24] described no major effects on fertility after 131-I treatment for childhood DTC, it cannot be taken as a superficial implication of administering such therapy without restriction in young women. Several data seem to confirm that 131-I does not increase the risk of congenital abnormalities in offspring of DTC survivors [38]. Other adverse effects on salivary glands and bone marrow suppression have been noted with multiple or higher doses of 131-I [38].

For women who survived a childhood neuroblastoma, Utriainen et al. [27] showed that the risk of POI was determined by total body irradiation (TBI) since almost every woman subjected to TBI was affected by such pathology. In this scenario, POI was likely to happen after spontaneous puberty. It is important to remember that even patients with spontaneously developed premature menopause needed MHT already a few years after menarche, which is significantly related to a severely reduced ovarian reserve [29].

Roshandel et al. [26] found that female CCS treated with RT showed lower AMH and inhibin B levels with a reduced AFC compared to controls. Similar to neuroblastoma survivors, there were reduced levels of AMH, inhibin B, and AFC when a TBI regimen was used. Otherwise, AMH levels and subsequent fertility were similar to the age-matched healthy controls [26].

Van der Kooi et al. [28] investigated the longitudinal decline of gonadal function in female CCS with a very long-term follow-up. Even though there was an initial decline in the AMH levels, the further decline in AMH levels in long-term female CCS was not accelerated. For this reason, at the end of a follow-up of at least 10 years, AMH levels were not different between female CCS and healthy controls [28].

This systematic review and network meta-analysis has several limitations. First, the number of qualified papers was relatively small, with small sample sizes. Second, additional outcomes and subgroup analysis were not evaluated due to retrieved data from original studies, since only two studies performed subgroup analyses according to the subtype of therapy. Although all the included trials followed strict protocols, for most papers, data were retrospectively collected, leading to possible biases. However, due to the nature of the disease, it would be impossible to perform randomized trials; for this reason, such research should be considered the best evidence that can be achieved.

In fact, a strength of our study is the quality of selected papers, since only papers with an overall low risk of bias were included in the network meta-analysis. 

Another strength of the study is the wide range of geographical areas covered by the available research, extending the possible generalization of the results. An additional robustness of this quantitative synthesis is the inclusion of only age-matched cohort studies, avoiding confounders of purely observational studies without controls.

## 5. Conclusions

In conclusion, AMH represents a valuable method for evaluating the ovarian reserves in female CCS. According to this network meta-analysis, there were no significant differences between treated CCS and healthy controls for every subtype of childhood cancer and no differences between indirect comparisons of evaluated treatments. However, according to the SUCRA analysis, neuroblastoma survivors during infancy seem more prone to have impaired AMH levels, followed by unselected female CCS. The risk of developing a POI is, indeed, notable in every CCS, since direct and indirect comparisons showed no differences between subtypes of cancer treatments. In addition, SUCRA analysis showed that such risk should be considered especially when total body or localized RT is performed and in the case of a childhood neuroblastoma. However, further comparative studies between different therapeutic strategies are needed to compare therapeutic regimens, doses, as well as RT subtypes in female CCS to avoid the risk of impaired ovarian reserves and POI.

## Figures and Tables

**Table 1:** Main characteristics of the studies included in the quantitative synthesis.

Study	Year	Design	Duration	Location	Population	Sample Size (Interventions vs. Controls)	Interventions	Controls	Outcomes
Nyström [25]	2018	Retrospective case–control	1964 to 2008	Sweden	Unselected female CCS	331 (167 vs. 164)	Surgery only RT (TBI or cranial) CT (alkylating agents) HSCT	Healthy age-matched	AMH levels AFC Ovarian volume Inhibin B levels FSH levels E2 levels
Nies [24]	2019	Retrospective case–control	1970 to 2013	Netherlands	Unselected female CCS	114 (57 vs. 57)	131-I	Healthy age-matched	AMH levels
Bath [22]	2003	Retrospective	NA	UK	Unselected female CCS	22 (11 vs. 11)	CT and/or RT	Healthy age-matched	AMH levels FSH levels Inhibin B levels
Harzif [23]	2020	Prospective Cross-sectional	May 2015 to December 2017	Indonesia	Unselected female CCS	88 (44 vs. 44)	Surgery CT and/or RT	Healthy age-matched	AMH levels
Roshandel [26]	2021	Retrospective case–control	1970 to 2013	Netherlands	Female ALL survivors	125 (67 vs. 58)	CT only vs CT and RT	Healthy age-matched	AMH levels
Utriainen [27]	2019	National cohort	1980 to 2000	Finland	Female neuroblastoma survivors	40 (20 vs. 20)	CT (alkylating agents) And HSCT	Healthy age-matched	AMH levels
van der Kooi [28]	2016	Retrospective single center cohort	1960 to 2005	Netherlands	Unselected female CCS	192 (96 vs. 96)	CT and/or RT	Healthy age-matched	AMH levels, AMH decrease
Thomas-Teinturier [8]	2015	Prospective cross-sectional	NA	France	Unselected female CCS	125 (105 vs. 20)	CT (alkylating agents) RT (subdiaphragmatic)	Healthy age-matched	AMH levels AFC

Abbreviations. CCS: childhood cancer survivors; CT: chemotherapy; RT: radiotherapy; ALL: acute lymphoid leukemia; AMH: anti-Mullerian hormone; AFC: antral follicle count; HSCT: hematopoietic stem cells transplant; NA: not available.

**Table 2:** Inclusion and exclusion criteria used by the authors of the original studies to define the sample size.

Study	Inclusion Criteria	Exclusion Criteria
Nyström [25]	Women treated for childhood cancer below 18 years of age at diagnosis and had completed treatment more than two years before inclusion.	Women with rare tumors or with focal tumors outside of the central nervous system treated with surgery only
Nies [24]	Female survivors of childhood differentiated thyroid cancer, diagnosed at ≤18 years of age	Short follow-up time (less than 5 years) or other malignancies diagnosed before or after the diagnosis of thyroid cancer
Bath [22]	Women aged >16 years with more than 2 years since the end of therapy	Women with irregular or absent menstrual cycle and elevated gonadotrophins before tumor diagnosis; active treatment with chemotherapy or radiotherapy or if they did not sign informed consent.
Harzif [23]	Women aged >18 years with treated for adolescence or early reproductive age cancer	Incomplete informed consent.
Roshandel [26]	NA	NA
Utriainen [27]	Female survivors of high-risk neuroblastoma	NA
van der Kooi [28]	Survivors with diagnosis of a primary tumor between 1960 and 2005 and in complete remission	NA
Thomas-Teinturier [8]	Women who received alkylating agents during childhood for a sarcoma, neuroblastoma, lymphoma, acute leukemia, or other tumors with 0.3 years follow-up after the end of treatment	Women diagnosed with ovarian tumors, brain or pelvic radiotherapy, or treatment with busulfan or thiotepa.

Abbreviations. NA: not available.

**Table 3:** Main findings reported in the original studies included in the quantitative synthesis.

Study	Sample Size	Mean AMH Levels (SD)		*p*-Value	POI Incidence (*n*/N)		*p*-Value
	(Intervention vs. controls)	Intervention	Controls		Intervention	Controls	
Nyström [25]	331 (167 vs. 164)	0.4 (0.4)	1.4 (0.4)	<0.01	20/167	0/164	<0.01
Nies [24]	114 (57 vs. 57)	2.0 (1.0)	1.6 (0.9)	0.29	0/57	0/157	NS
Bath [22]	22 (11 vs. 11)	1.3 (0.3)	2.1 (0.4)	<0.05	NA	NA	NA
Harzif [23]	88 (44 vs. 44)	1.1 (3.0)	3.9 (2.0)	<0.01	NA	NA	NA
Roshandel [26]	125 (67 vs. 58)	NA	NA	NA	8/67	1/58	<0.01
Utriainen [27]	40 (20 vs. 20)	0.2 (0.2)	1.7 (0.6)	<0.01	6/20	0/20	<0.01
van der Kooi [28]	192 (96 vs. 96)	2.5 (0.5)	2.5 (0.5)	0.68	NA	NA	NA
Thomas-Teinturier [8]	125 (105 vs. 20)	1.1 (1.0)	2.2 (2.0)	<0.01	2/102	0/20	NS

Abbreviations: NA: not available; NS: not significant.

## Supplementary Materials

The following are available online at https://www.mdpi.com/article/10.3390/cancers13246331/s1: Table S1: Detailed study search queries; Table S2: Quality assessment using Newcastle-Ottawa Scale. Table S3: SIDE analysis for mean AMH levels; Table S4: SIDE analysis for POI incidence; Supplement File S1: PRISMA-NMA checklist.
