# Supplementary material for: Serum Anti-Müllerian Hormone Levels and Risk of Premature Ovarian Insufficiency in Female Childhood Cancer Survivors: Systematic Review and Network Meta-Analysis
Source: Cancers (Basel). 2021 Dec 16;13(24):6331. doi: 10.3390/cancers13246331 (PMC8699404; doi:10.3390/cancers13246331)
Supplement: Supplementary file 1 [file cancers-13-06331-s001.zip › cancers-1464436-supplementary/cancers-1464436-for conversion-supp/Tables_S3_S4.pdf]

Tables S3 and S4

Table S3. SIDE analysis for mean AMH levels

Side	Direct	Indirect	Difference	Coef.	Std. Err. Coef.	Std. Err. Coef.	Std. Err. P>|z|
A C *	.8821219	.4840755	.2056847	227.9047	.6764372	227.9052	0.998
B C *	1.5	1.121028	1.764238	695.8296	-.2642384	695.8308	1.000
C D *	.4	1.042334	-1.76493	599.6303	2.16493	599.6312	0.997

Table S4. SIDE analysis for POI incidence

Side	Direct	Indirect	Difference	Coef.	Std. Err. Coef.	Std. Err. Coef.	Std. Err. P>|z|
A D *	-2.044956	2.475975	-1.88636	658.7511	-.1585956	658.7558	1.000
B D *	-1.973389	1.90217	-2.634181	2191.441	.6607922	.6607922	1.000
C D *	-2.911226	2.691189	-2.790475	1700.67	-.120751	1700.673	1.000
D E *	-4.32e-10	3.00093	4.089859	6870.333	-4.089859	6870.334	1.000
