# Supplementary material for: Serum Anti-Müllerian Hormone Levels and Risk of Premature Ovarian Insufficiency in Female Childhood Cancer Survivors: Systematic Review and Network Meta-Analysis
Source: Cancers (Basel). 2021 Dec 16;13(24):6331. doi: 10.3390/cancers13246331 (PMC8699404; doi:10.3390/cancers13246331)
Supplement: Supplementary file 1 [file cancers-13-06331-s001.zip › cancers-1464436-supplementary/cancers-1464436-for conversion-supp/Table_S1.pdf]

Table S1. Detailed study search queries

**MEDLINE (accessed through PubMed)**
("AMH"[All Fields] OR ("anti mullerian hormone"[MeSH Terms] OR ("anti mullerian"[All Fields] AND "hormone"[All Fields]) OR "anti mullerian hormone"[All Fields] OR ("anti"[All Fields] AND "mullerian"[All Fields] AND "hormone"[All Fields]) OR "anti mullerian hormone"[All Fields])) AND (("femal"[All Fields] OR "female"[MeSH Terms] OR "female"[All Fields] OR "females"[All Fields] OR "female s"[All Fields] OR "femals"[All Fields]) AND ("cancer survivors"[MeSH Terms] OR ("cancer"[All Fields] AND "survivors"[All Fields]) OR "cancer survivors"[All Fields]))
**EMBASE**
(amh OR 'anti-mullerian hormone'/exp OR 'anti-mullerian hormone' OR ('anti mullerian' AND ('hormone'/exp OR hormone))) AND ('female cancer survivors' OR (('female'/exp OR female) AND ('cancer'/exp OR cancer) AND ('survivors'/exp OR survivors)))
**SCOPUS**
TITLE-ABS-KEY ((amh OR anti-mullerian AND hormone OR anti Mullerian hormone) AND female AND cancer AND survivors)
**LILACS / Scielo.br**
(AMH or anti-mullerian hormone) and (female cancer survivors)
**COCHRANE AT CENTRAL**
(AMH or anti-mullerian hormone) and (female cancer survivors)
**CINAHL / PsycINFO / AMED / PsycExtra (accessed through EBSCO – IDEM for Italian Universities)**
(AMH or anti-mullerian hormone) and (female cancer survivors) AND Cerca anche nel testo completo degli articoli; Applica argomenti equivalenti
**Clinicaltrials.gov / ICTRP (accessed through CENTRAL)**
((AMH or anti-mullerian hormone) and (female cancer survivors)):ti,ab,kw
