# Supplementary material for: Serum Anti-Müllerian Hormone Levels and Risk of Premature Ovarian Insufficiency in Female Childhood Cancer Survivors: Systematic Review and Network Meta-Analysis
Source: Cancers (Basel). 2021 Dec 16;13(24):6331. doi: 10.3390/cancers13246331 (PMC8699404; doi:10.3390/cancers13246331)
Supplement: Supplementary file 1 [file cancers-13-06331-s001.zip › cancers-1464436-supplementary/cancers-1464436-for conversion-supp/Table_S2_NOS.pdf]

Table S3. Quality scores of the studies included in the meta-analysis, assessed by the Newcastle-Ottawa scale.

		**Selection**				**Comparability [a]**	**Outcome**			**Overall quality**
**Author**	**Year**	**Representativeness of the exposed cohort**	**Selection of the non-exposed cohort**	**Ascertainment of exposure**	**Demonstration that the outcome of interest was not present at the start of the study**	**Comparability of cohorts on the basis of the design or analysis**	**Assessment of the outcome**	**Was follow-up enough for outcomes to occur**	**Adequacy of follow-up of cohorts**	
Nyström	2018	*	*	*	*	**	*	*	*	**9**
Nies	2019	*	*	*	*	**	*	*	*	**9**
Bath	2003	0	*	*	*	*	*	0	*	**6**
Harzif	2020	*	*	*	*	**	0	*	*	**8**
Roshandel	2021	*	*	0	*	**	*	*	*	**8**
Utriainen	2019	*	*	*	*	**	*	*	*	**8**
van der Kooi	2016	*	*	0	*	**	0	*	*	**7**
Thomas-Teinturier	2015	*	*	0	*	**	0	*	*	**7**

Newcastle-Ottawa scale for assessment of quality of included studies - cohort studies (each asterisk represents if individual criterion within the subsection was fulfilled). [a] Comparability of cohorts: for the most important factor: study controls adjusted for age; additional factor: study controls for BMI
